# A multi-method spatial analysis of dysentery incidence determinants across Chinese provinces

**DOI:** 10.3389/fpubh.2025.1663473

**Published:** 2025-10-23

**Authors:** Ke Hu, Xingjin Yang, Shuiping Ou, Chaojie Li, Xing Zhang, Di Xiao, Mingyang Yu

**Affiliations:** ^1^Department of Scientific Research and Teaching, Xiamen Haicang Hospital, Xiamen, Fujian, China; ^2^QianDongNanZhou Center for Disease Control and Prevention, QianDongNanZhou, Guizhou, China; ^3^Honwing Pharma(Guizhou) Company Limited, QianDongNanZhou, Guizhou, China; ^4^Xingtai Center for Disease Control and Prevention, Xingtai, Hebei, China; ^5^Nanjing Lishui Dongping Street Health Center, Nanjing, Jiangsu, China; ^6^Community Health Service Center of Jiuxian Tongliang District, Chongqing, China; ^7^Hypertension Prevention and Control Center, Fuwai Central China Cardiovascular Hospital, Zhengzhou, Henan, China

**Keywords:** dysentery incidence, influencing factors, multiple linear regression, spatial error model, geographically weighted regression, multiscale geographically weighted regression

## Abstract

**Introduction:**

Dysentery remains a significant notifiable Class B infectious disease in China, exhibiting distinct spatial variations in incidence patterns. This persistent geographical heterogeneity necessitates a systematic investigation into the underlying influencing factors to inform targeted prevention and control strategies.

**Methods:**

Our analytical approach incorporated Moran's I index for spatial autocorrelation analysis, multiple linear regression (MLR) for preliminary assessment, and advanced spatial regression models including spatial error model (SEM), geographically weighted regression (GWR), and multiscale geographically weighted regression (MGWR). The analysis incorporated socioeconomic, educational, healthcare, and demographic factors within a unified spatial framework.

**Results:**

The analysis revealed three key findings: (1) Significant spatial clustering of dysentery incidence with identified high-risk concentration in the Beijing-Tianjin region; (2) Superior performance of MGWR modeling in capturing spatial heterogeneity compared to conventional methods; (3) Distinct regional variations in dominant factors, with economic development most influential in western China, education factors predominant in northeastern areas, and healthcare resource availability showing strongest impact in the northeast but minimal effect in southern regions.

**Conclusions:**

The study demonstrates the value of multiscale spatial analysis in understanding geographical disease patterns, revealing that dysentery incidence in China is governed by different factors across regions.

## 1 Introduction

As a notifiable Class B infectious disease in China, dysentery shows pronounced spatial aggregation and geographical disparities in incidence rates. Nationwide epidemiological surveillance data reveal pronounced regional aggregation, with persistently high-incidence areas centered on the Beijing-Tianjin-Hebei urban cluster (1, 2), while provinces like Hunan and Zhejiang in the Yangtze River basin also demonstrate elevated disease risk (3, 4). This spatial heterogeneity manifests more precisely at finer scales, evidenced by significant local hotspots such as the Hangzhou Bay urban belt in Zhejiang (4) and mountainous areas of western and southern Hunan (3). Such complex spatial patterns not only reflect the multidimensional transmission mechanisms but also underscore the necessity for investigating underlying determinants.

Existing studies have revealed multidimensional driving factors influencing the spatial distribution of dysentery. At the socioeconomic level, per capita GDP demonstrates significant explanatory power (5), likely attributable to improved sanitation infrastructure and enhanced healthcare accessibility resulting from economic development. The impact of educational level is particularly prominent. Studies have shown that individuals with lower educational attainment face a significantly higher risk of infection (6, 7). This association is even more pronounced among children (8), reflecting the critical role of health literacy in disease prevention.

The uneven distribution of medical resources also exerts considerable influence (5). However, population density has a dual effect: it can potentially facilitate transmission by increasing the frequency of contact (1, 2), while simultaneously generating protective effects through enhanced public health investments (9). Additionally, larger household size elevates risk by increasing close contact opportunities (10, 11), and sanitation disadvantages in low-income areas (1) coupled with environmental pressures from rapid urbanization (9) have been conclusively linked to disease prevalence. These associations are particularly evident in highly urbanized regions like the Beijing-Tianjin-Hebei area (1).

Methodologically, spatial econometrics offers a suite of innovative analytical techniques. While Multiple Linear Regression (MLR) identifies global risk factors, it fails to account for spatial autocorrelation (12, 13). Spatial lag models (SLM) incorporate spatial weight matrices to capture spillover effects from neighboring areas (14, 15), while spatial error models (SEM) address spatial dependence in the error terms (16, 17). Geographically weighted regression (GWR) advances the field by relaxing spatial stationarity assumptions to reveal local spatial heterogeneity (18, 19). The more recent Multiscale Geographically Weighted Regression (MGWR), which allows variable-specific bandwidths, has demonstrated superior performance in provincial-scale analyses (20, 21), by simultaneously identifying both broad-scale and localized determinants (22, 23).

It is precisely to address the complex and multiscale nature of dysentery's spatial drivers that this study develops a multidimensional approach. Building upon this methodological foundation, we construct an integrated spatial analytical framework that systematically combines MLR, SEM, SLM, GWR, and MGWR. This comprehensive strategy is designed to disentangle the intricate spatial distribution patterns of dysentery incidence across China. By integrating global, local, and multiscale perspectives, the framework enables a more robust examination of the underlying driving mechanisms at the provincial level, thereby offering deeper insights for targeted public health interventions.

## 2 Methods

### 2.1 Data

Building upon the theoretical framework established in the background section and considering data availability, this study selected seven core influencing factor indicators (detailed in Table 1) covering multiple dimensions including economic development, education level, population structure, family structure, healthcare resources, consumption level, and urban-rural structure. The analysis units comprised 31 provincial-level administrative regions (including provinces, autonomous regions, and municipalities directly under the central government) of mainland China. All data were obtained from authoritative statistical sources: dysentery incidence data in 2022 were extracted from the China Health Statistical Yearbook, while other explanatory variables were sourced from the officially published China Statistical Yearbook.

**Table 1:** Key factors selected for analysis.

**Variable**	**Factors**
Economic development	GDP per capita
Education level	Number of college students per 100,000 persons
Healthcare resources	Number of hospital beds per 1,000 persons
Population structure	Population density
Family structure	Average household size
Consumption level	Household consumption expenditure per capita
Urban-rural structure	Urbanization rate

### 2.2 Spatial distribution characteristics analysis

The study first employed Geographic Information System spatial visualization techniques to construct a thematic map of dysentery incidence across 31 provincial-level administrative regions in mainland China. By transforming provincial incidence data into intuitive spatial distribution maps, the regional variations in disease incidence intensity were clearly presented.

### 2.3 Spatial autocorrelation analysis

This research adopted spatial autocorrelation analysis methods to systematically evaluate the spatial distribution characteristics of dysentery incidence from the following two dimensions.

#### 2.3.1 Global spatial autocorrelation analysis

The study employed Global Moran's I index to quantitatively assess the spatial autocorrelation of dysentery incidence rates. The mathematical expression is as follows (Equation 1) (24):


(1) $$\begin{array}{l} I=\frac{n\overset{n}{\underset{i=1}{\sum }}\overset{n}{\underset{j=1}{\sum }}W_{ij}\left(x_{i}-\bar{x}\right)\left(x_{j}-\bar{x}\right)}{\overset{n}{\underset{i=1}{\sum }}\overset{n}{\underset{j=1}{\sum }}W_{ij}\overset{n}{\underset{i=1}{\sum }}{\left(x_{i}-\bar{x}\right)}^{2}} \end{array}$$

Where *n* represents the sample size, *w*_ij_ denotes the elements of the spatial weight matrix, x_i_ and x_j_ are observed values, and $\bar{x}$ is the mean value. The spatial weight matrix is represented by the elements *w*_ij_.

The Moran's I index ranges from −1 to 1, where positive values (*I* > 0) indicate positive spatial autocorrelation (clustering of similar values), negative values (*I* < 0) represent negative spatial autocorrelation (dissimilar adjacent values), and values approaching zero (*I* ≈ 0) suggest random spatial distribution. All results were verified for statistical significance using *Z*-test (*p* < 0.05) (25).

#### 2.3.2 Local Moran's index (LISA)

The Local Indicators of Spatial Association (LISA) method was applied to examine spatial correlation patterns between provincial administrative regions and their neighboring areas. Using the same variable definitions as the global Moran's I, The computational formula is expressed as (Equation 2) (26):


(2) $$\begin{array}{l} I_{i}=\frac{n\left(x_{i}-\bar{x}\right)\overset{n}{\underset{j=1}{\sum }}W_{ij}\left(n_{j}-\bar{n}\right)}{\overset{n}{\underset{j=1}{\sum }}{\left(x_{j}-\bar{x}\right)}^{2}} \end{array}$$

The LISA analysis identified four characteristic spatial patterns: high-high clusters (HH, indicating adjacent high-incidence areas), low-low clusters (LL, indicating adjacent low-incidence areas), high-low outliers (HL, high-incidence areas surrounded by low-incidence areas), and low-high outliers (LH, low-incidence areas surrounded by high-incidence areas). All identified spatial patterns were statistically significant (*p* < 0.05) and were visualized through spatial mapping to effectively demonstrate the geographical variations in dysentery incidence (27).

### 2.4 Multiple linear regression (MLR)

The study employed MLR to analyze influencing factors of dysentery incidence, with the basic model formulation (Equation 3) (28):


(3) $$\begin{array}{l} Y=\beta_{0}+\beta_{1}X_{1}+\beta_{2}X_{2}+\ldots +\beta_{p}X_{p}+\varepsilon \end{array}$$

Where *Y* represents the incidence rate of dysentery, *X*1 through *X*_p_ denote the influencing factors, β0is the constant term, β1through β_p_ are the variable coefficients, and ε is the random error term, which follows a normal distribution with a mean of 0 and a variance of σ^2^. Model evaluation included *R*^2^ for explanatory power, Akaike Information Criterion (AIC) as well as the log-likelihood value for model comparison, and Variance Inflation Factors (VIF; threshold = 5) for multicollinearity assessment (29).

### 2.5 Global spatial regression models

#### 2.5.1 Spatial error model (SEM)

The SEM addresses spatial autocorrelation in regression residuals through its dual-component structure. The model specification consists of two components (Equations 4 and 5):


(4) $$\begin{array}{l} Y=X_{\beta }+u \end{array}$$

(5) $$\begin{array}{l} u=\lambda w_{u}+\varepsilon \end{array}$$

Where λ denotes the spatial error autocorrelation coefficient, and *W*_u_ represents the spatial lag effect of the error terms. By incorporating spatial dependence in error terms, SEM effectively resolves estimation bias caused by ignored spatial autocorrelation in conventional regression (30).

#### 2.5.2 Spatial lag model (SLM)

The SLM characterizes spatial interaction effects through a spatially lagged dependent variable: its mathematical formulation is expressed as (Equation 6) (31):


(6) $$\begin{array}{l} Y=\rho W_{Y}+X_{\beta }+\varepsilon \end{array}$$

Where ρ quantifies the intensity of spatial dependence, *W* defines spatial relationships via the weight matrix, *W*_Y_ represents the weighted average influence from neighboring areas, *X* is the matrix of explanatory variables, β denotes the coefficient vectors, and ε is the random error term. This model accounts for spatial spillover effects, overcoming limitations of traditional regression that neglect spatial dependence.

### 2.6 Local spatial regression models

#### 2.6.1 Geographically weighted regression (GWR)

GWR represents an innovative spatial analysis approach that addresses the limitation of spatially invariant parameters in traditional regression models, effectively revealing spatial heterogeneity in variable relationships. The model incorporates spatial location information into the regression equation, allowing coefficients to vary geographically. Its basic formulation is expressed as (Equation 7) (26):


(7) $$\begin{array}{l} Y_{i}=\beta_{0}\left(u_{i},v_{i}\right)+\overset{m}{\underset{k=1}{\sum }}\beta_{k}\left(u_{i},v_{i}\right)x_{ik}+\varepsilon_{i} \end{array}$$

Where (*u*_i_, *v*_i_) denotes the spatial coordinates of sample points and β_k_ (*u*_i_, *v*_i_) represents location-specific regression coefficients. The model employs Gaussian kernel functions and distance-decay methods to construct spatial weight matrices, estimates parameters through weighted least squares, and optimizes bandwidth selection using cross-validation (CV) techniques. Compared with conventional methods, GWR provides more precise characterization of spatial variations in explanatory variable effects, offering refined analytical capabilities for spatial data.

#### 2.6.2 Multiscale geographically weighted regression (MGWR)

MGWR, as an important extension of GWR, introduces variable-specific bandwidth parameters (*b*_wk_) to differentially characterize the spatial scales of various explanatory variables. Its basic formulation is Equation 8:


(8) $$\begin{array}{l} Y_{i}=\beta_{0}\left(u_{i},v_{i},bw_{0}\right)+\overset{m}{\underset{k=1}{\sum }}\beta_{k}\left(u_{i},v_{i},bw_{k}\right)x_{ik}+\epsilon_{i} \end{array}$$

Where each explanatory variable *x*_ik_ corresponds to an independent bandwidth parameter *b*_wk_. This design enables more accurate capture of spatial heterogeneity in each variable's influence range. For parameter estimation, MGWR employs an iterative back-fitting algorithm for optimization, determines optimal bandwidth combinations through CV criteria, and constructs spatial weight matrices based on Gaussian kernel functions (32). Compared with conventional GWR, key advantages of MGWR include: (1) effectively addressing parameter estimation bias caused by single bandwidth through variable-specific bandwidth settings; (2) precisely identifying differential spatial influence ranges among explanatory variables; and (3) significantly improving model accuracy in analyzing complex spatial dependence relationships. These characteristics establish MGWR as a more reliable and precise methodological tool for spatial heterogeneity analysis, particularly suitable for research scenarios where explanatory variables exhibit varying spatial scales of influence.

### 2.7 Software

This study adopted a multi-platform collaborative approach for spatial statistical analysis. Spatial data processing and visualization were conducted using ArcGIS 10.2 software, encompassing analytical procedures such as spatial autocorrelation testing, and multicollinearity diagnostics. The construction and parameter estimation of conventional regression and spatial econometric models (SLM/SEM) were performed in the GeoDa 1.22 environment, while the analysis of GWR and its multiscale extension (MGWR) was implemented using the specialized MGWR2.2 software. The foundational geographic data were sourced from the National Platform for Common Geospatial Information Services [Map Review No.: GS (2024)0650], with all statistical tests employing two-tailed testing at a significance level of α = 0.05.

## 3 Results

### 3.1 Descriptive and spatial autocorrelation analysis results

**Figure 1:**
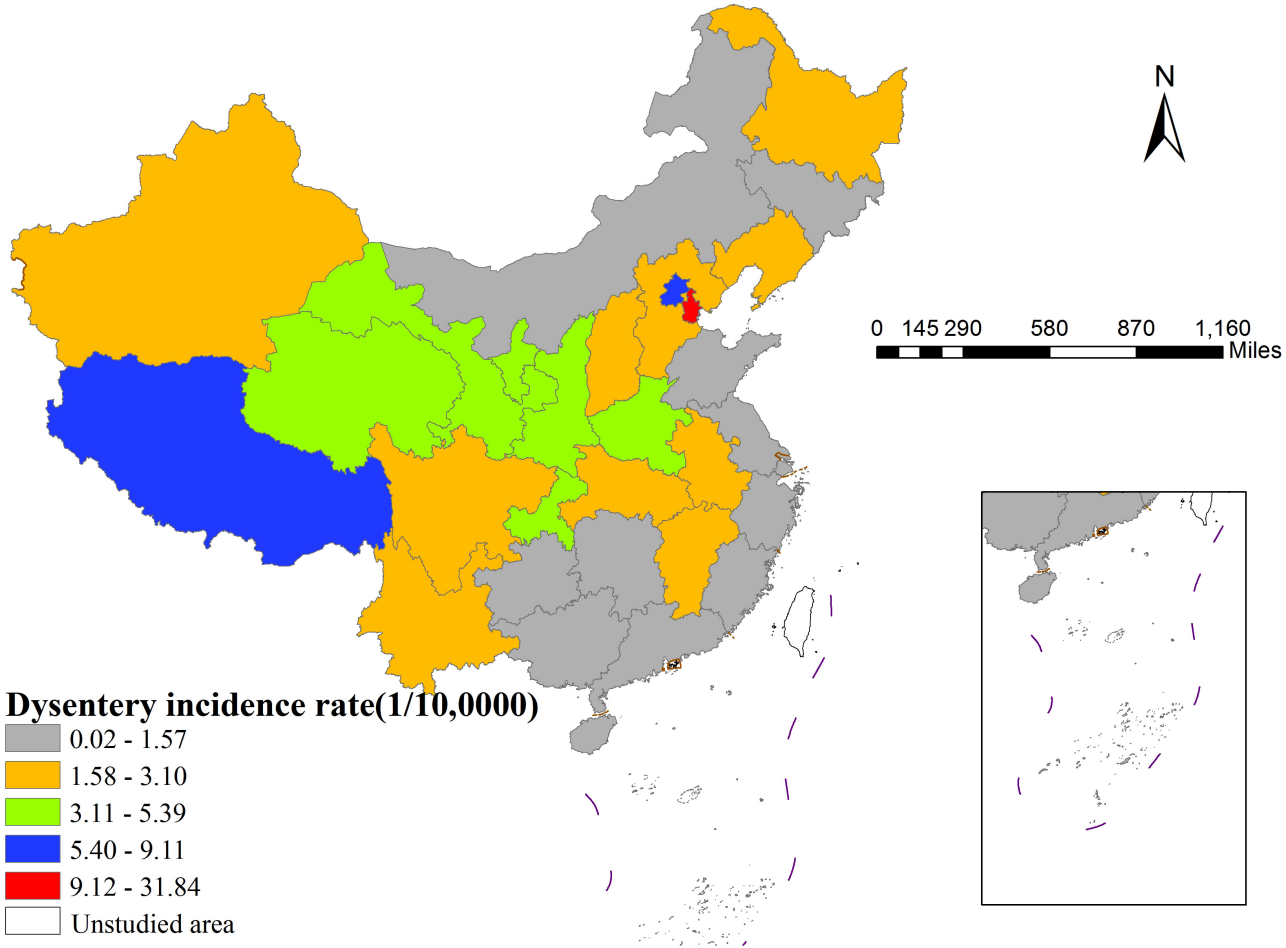
The spatial distribution of dysentery incidence in China (2022).

**Figure 2:**
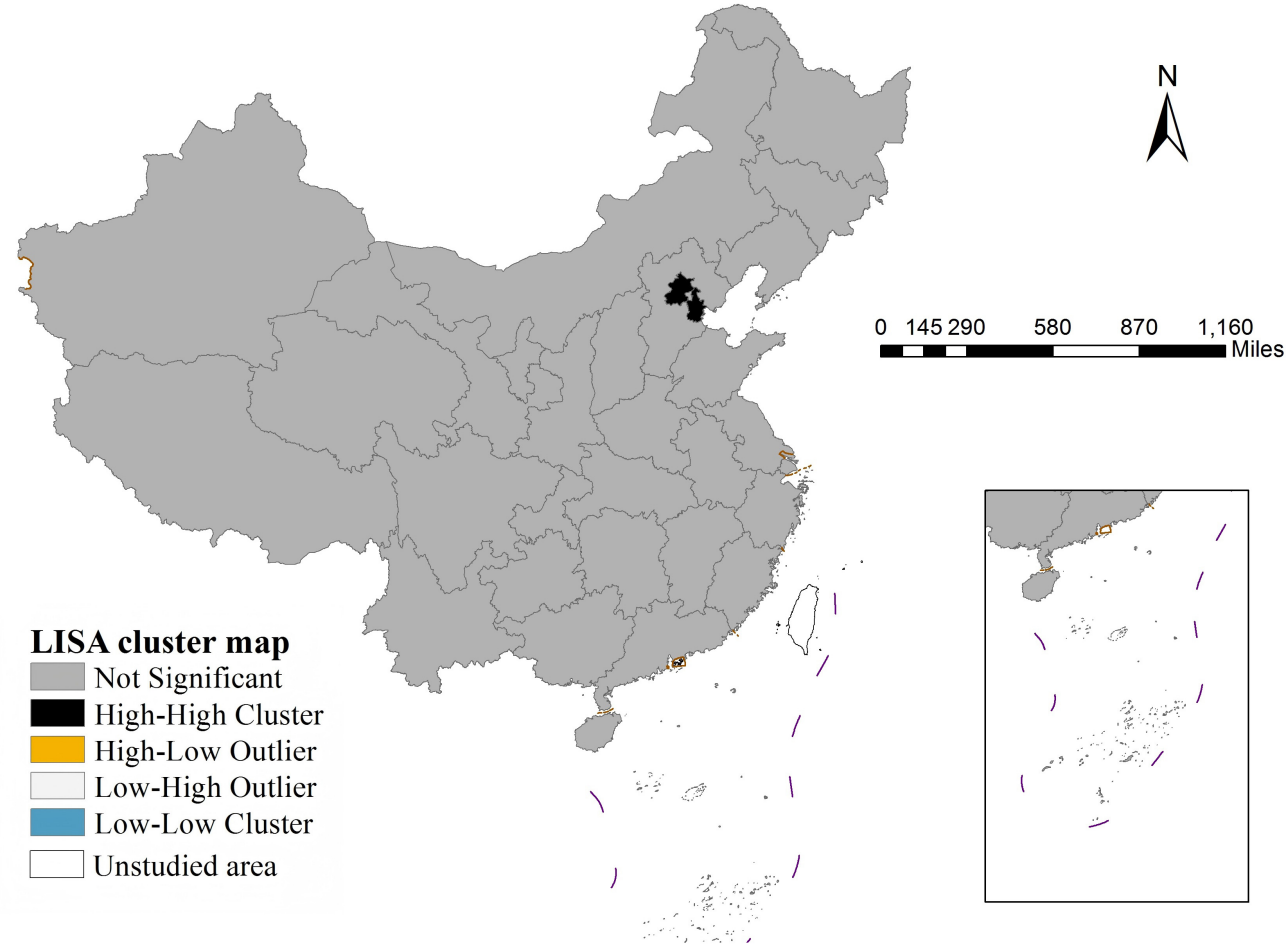
LISA cluster map of dysentery incidence (2022).

As shown in Figure 1, there was significant spatial heterogeneity in the incidence of dysentery across Chinese provinces in 2022. Geographically, Tianjin recorded the highest incidence rate nationwide at 31.84 per 100,000, while Shanghai had the lowest rate (0.02 per 100,000). Moran's I index analysis (*I* = 0.203, *p* < 0.001) indicated a significant spatial clustering pattern in dysentery incidence. LISA cluster analysis (Figure 2) identified specific local patterns, most notably a high-high cluster centered in the Beijing-Tianjin region, demonstrating clear spatial aggregation and geographical disparities in disease distribution.

### 3.2 Results of MLR

Results In the preliminary analysis phase, this study employed VIF to diagnose multicollinearity among explanatory variables. The results revealed significant multicollinearity between household consumption expenditure per capita, urbanization rate, and other variables (VIF > 9). After excluding these two variables, the VIF values of the remaining variables all fell within an acceptable range (VIF < 4).

Using the optimized variable set, MLR analysis identified only two statistically significant predictors: the number of college students per 100,000 persons and the number of hospital beds per 1,000 persons (Table 2). Notably, spatial autocorrelation testing of the model residuals reached a significant level (*p* = 0.01), providing a theoretical basis for subsequent adoption of spatial regression models (Table 3).

**Table 2:** Results of the SEM and MLR.

**Model fit**	**SEM**		**MLR**	
	**Coefficient**	***p*** **-Value**	**Coefficient**	***p*** **-Value**
Intercept	−0.0387	0.8691	−0.0000	1.0000
GDP per capita	−0.3917	0.0961	−0.2593	0.3999
Number of college students per 100,000 persons	0.4164	0.0185	0.4584	0.0324
Number of hospital beds per 1,000 persons	−0.5395	0.0022	−0.4742	0.0288
Population density	0.0780	0.6888	0.0262	0.9183
Average household size	−0.2117	0.3138	−0.0558	0.8030

**Table 3:** Goodness-of-fit of the four models.

**Model fit**	**MGWR**	**GWR**	**SEM**	**MLR**
Moran's I	−0.072 (*p* = 0.705)	0.189 (*p* = 0.035)	−0.024 (*p* = 0.927)	0.190 (*p* = 0.01)
*R* ^2^	0.584	0.318	0.392	0.305
AIC	79.874	90.317	84.959	87.682
Log likelihood	−30.391	−38.066	−36.480	−37.841

### 3.3 Results of global spatial regression models

This study constructed SLM and SEM for analysis. The results showed that the spatial error coefficient (λ = 0.428, *p* = 0.035) was more significant than the spatial lag coefficient (*W*_Y_ = 0.34, *p* = 0.099), indicating that the SEM was more suitable for this study.

After controlling for spatial effects, both the number of college students per 100,000 persons and the number of hospital beds per 1,000 persons remained statistically significant (*p* < 0.05), with their significance further enhanced compared to MLR (Table 2). Model comparisons revealed that the SEM exhibited improved *R*^2^, higher log-likelihood values, and lower AIC, confirming its superiority (Table 3).

Residual tests indicated that the SEM effectively eliminated spatial autocorrelation, addressing the spatial dependence bias inherent in traditional regression models and ensuring the reliability of the research findings (Table 3).

### 3.4 Results of local spatial regression models

This study systematically compared model fitting performance by constructing both GWR and MGWR. The results demonstrated that the MGWR model exhibited superior explanatory power regarding the relationship between dysentery incidence and influencing factors, with significantly better fitting performance than both GWR and SEM. Notably, the GWR model not only underperformed SEM in fitting effectiveness but also showed significant residual spatial autocorrelation (Table 3). Consequently, this study ultimately adopted the MGWR model with its multiscale analytical advantages for in-depth analysis.

The MGWR model results revealed that among the five core explanatory variables, three demonstrated statistically significant impacts on dysentery incidence (*p* < 0.05) with distinct spatial heterogeneity characteristics.

**Figure 3:**
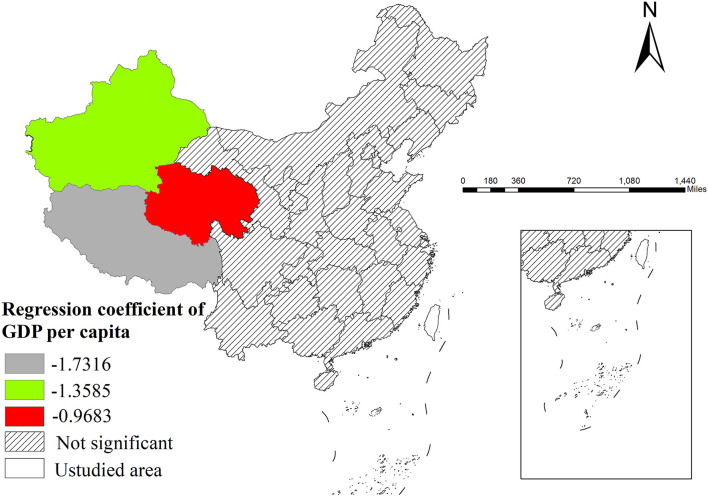
The spatial distribution of regression coefficient of GDP per capita.

Firstly, per capita GDP showed significant negative correlation with incidence only in western China, while its effect was nonsignificant in central and eastern regions (Figure 3).

**Figure 4:**
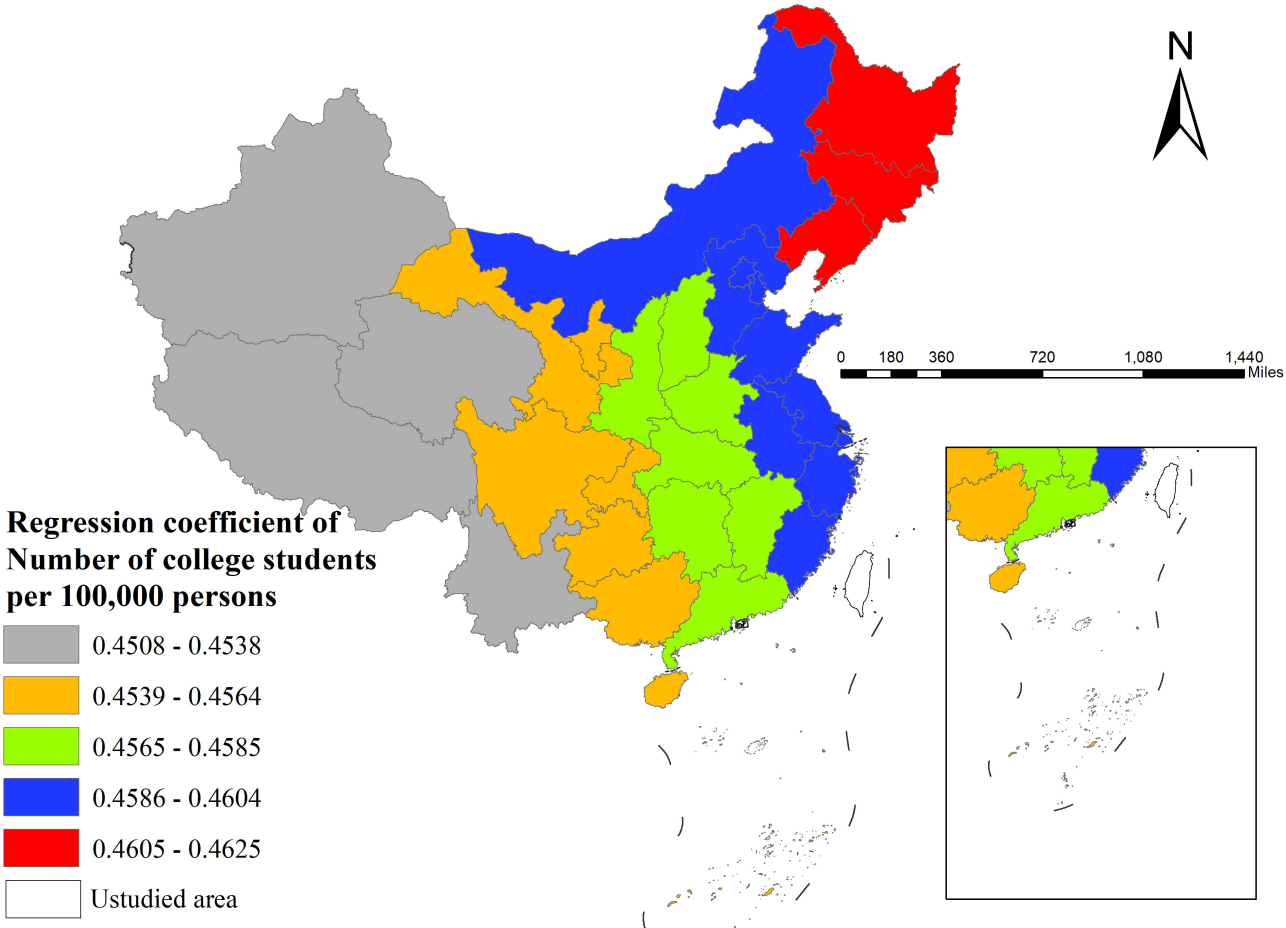
The spatial distribution of regression coefficient of number of college students per 100,000 persons.

Secondly, the number of college students per 100,000 persons exhibited positive effects, most pronounced in eastern areas (particularly in northeastern provinces) and relatively weaker in western regions (especially Xinjiang and Tibet; Figure 4).

**Figure 5:**
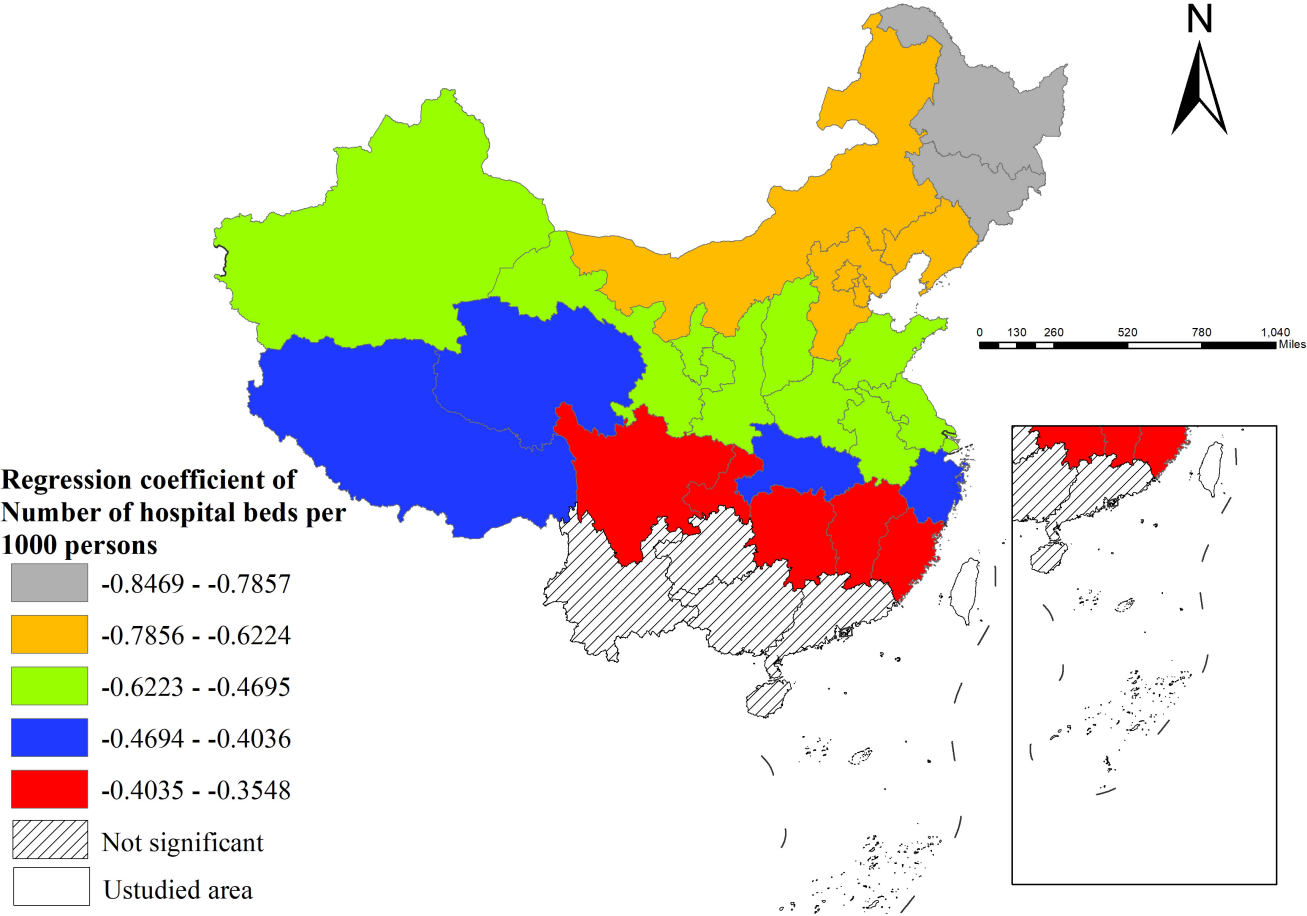
The spatial distribution of regression coefficient of number of hospital beds per 1,000 persons.

Thirdly, regarding healthcare resources, hospital beds per 1,000 persons demonstrated significant negative effects, particularly prominent in northeastern China, while southern regions (e.g., Guangdong, Guangxi, Yunnan, Guizhou) showed no statistically significant impact (Figure 5).

However, neither population density nor average household size showed statistically significant effects on dysentery incidence across all regions.

**Figure 6:**
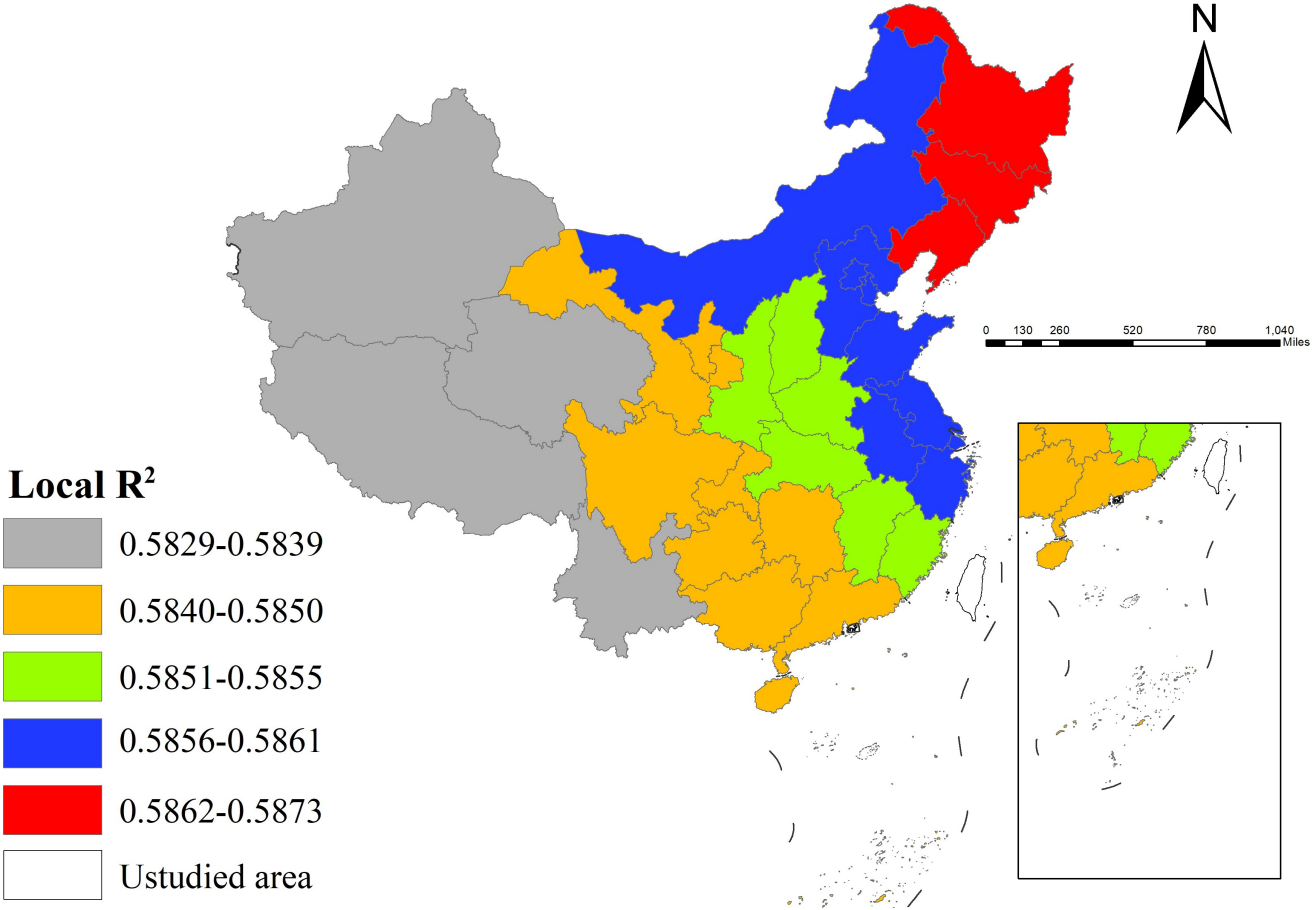
The spatial distribution of local *R*^2^.

Further spatial analysis indicated superior model fit in eastern regions (e.g., northeastern provinces, Beijing, Tianjin), where local *R*^2^ values were generally higher, suggesting strong explanatory power of selected variables for incidence variation. However, the model showed relatively poorer performance in western provinces (e.g., Yunnan, Tibet), implying potential needs for incorporating additional influencing factors to enhance explanatory capability in these areas (Figure 6).

### 3.5 Sensitivity analysis

The sensitivity analysis revealed that bandwidth selection critically influenced MGWR model outcomes. Comparative evaluation of MGWR models constructed under AIC_c_ and CV criteria (Table 4) showed that while the AIC_c_-based model achieved slightly better goodness-of-fit, its residuals still exhibited significant spatial autocorrelation. Therefore, the CV-based MGWR model demonstrated better robustness and proved more suitable for this study's data analysis.

**Table 4:** Comparison of model fit between AIC_c_ and CV criteria in MGWR.

**Model fit**	**MGWR (CV)**	**MGWR (AIC_c_)**
Moran's I	−0.072 (*p* = 0.705)	−0.370 (*p* = 0.003)
*R* ^2^	0.584	0.836
AIC	79.874	57.669
Log likelihood	−30.391	−15.957

## 4 Discussion

This study established a comprehensive analytical framework for dysentery incidence using four methodological approaches: MLR, SEM, GWR, and MGWR. The MLR model first identified key influencing factors; the SEM model confirmed the significant role of spatial dependence; the GWR model revealed the spatial heterogeneity characteristics of influencing factors; and finally, the MGWR model further enhanced the model's explanatory power and predictive accuracy through multiscale analysis. This sequential analysis not only elucidated the mechanisms influencing dysentery incidence but also identified regional variations in these factors, providing a scientific basis for targeted prevention and control measures.

Empirical results from the MGWR analysis GDP per capita demonstrated a significant negative effect on dysentery incidence in western China. This finding aligns with the conclusions of Zhan et al. (33), confirming the positive role of economic development in disease prevention and control. Potential mechanisms include: economically developed regions possess more robust healthcare resource allocation (5); and higher GDP levels facilitate improvements in public health infrastructure (e.g., water supply and sewage systems) (34). Notably, this negative effect is particularly pronounced in western regions with relatively weaker infrastructure, whereas in eastern regions, where baseline sanitary conditions are already better, the marginal effect of economic factors appears more limited.

The study also revealed the number of college students per 100,000 persons exerted a significant positive on disease incidence. Existing research (5, 35) suggests that the communal living patterns of college students (e.g., sharing tableware and sanitation facilities) may increase disease transmission risks. This effect is particularly pronounced in northeast regions with high concentrations of universities, likely due to higher population density and increased contact frequency.

Hospital beds availability showed a negative effect on dysentery incidence. Increased bed numbers may enhance healthcare accessibility, enabling timely hospitalization of patients and thereby reducing transmission risks (36). The inhibitory effect of hospital beds on dysentery incidence was stronger in northeastern China, which can be attributed to several region-specific factors: relative bed shortages, weaker primary healthcare systems, population outflow alleviating actual medical pressure, and potentially more effective inpatient isolation during cold weather conditions.

Notably, population density showed no significant influence on dysentery incidence. Previous studies (9) suggest this may reflect how robust public health interventions (e.g., vaccination programs and sanitation infrastructure) in high-density areas effectively counteract the potential risks of population aggregation. Additionally, research (37) has demonstrated that hot and humid climates significantly increase dysentery incidence–such strong climatic effects may overshadow the influence of population density.

Additionally, household size showed no statistically significant effect on dysentery incidence. This finding aligns with previous research (38), potentially because other stronger determinants like sanitation conditions may have overshadowed the role of household size.

Methodologically, this study contributes to the field by innovatively integrating multiple advanced spatial econometric approaches—including MLR, SEM, GWR, and MGWR—to establish a comprehensive analytical framework for identifying determinants of dysentery incidence. This multi-model synergistic approach helps overcome the limitations associated with traditional single-model analyses. By effectively capturing multiscale spatial heterogeneity, the framework provides a useful methodological reference for health geography research and supports the development of regionally tailored public health strategies.

Nevertheless, several limitations should be acknowledged: First, regarding data, the use of provincial-level analysis units due to official statistics availability constraints may insufficiently capture finer-scale (e.g., county/city-level) spatial variations. Meanwhile, the exclusion of certain important explanatory variables (e.g., environmental factors like temperature and humidity) due to data unavailability might partially compromise the comprehensiveness of findings. Second, methodologically, while the cross-sectional design effectively reveals spatial association patterns, it carries inherent limitations in causal inference. Finally, the models have not accounted for temporal dynamics of key factors like population mobility and prevention policies, which might affect in-depth understanding of dysentery transmission mechanisms.

## 5 Conclusion

This study systematically investigated the spatial distribution patterns and influencing factors of dysentery incidence in China using multiscale spatial analysis methods. The findings reveal significant spatial heterogeneity in dysentery incidence across China, with a distinct high-incidence cluster identified in the Beijing-Tianjin region. Methodologically, MGWR demonstrated superior analytical performance compared to traditional spatial regression approaches, enabling more precise identification of the spatial variation characteristics of various influencing factors.

Substantial regional differences in dominant factors affecting dysentery incidence. Economic development level showed significant influence in western regions, while educational factors played a particularly prominent role in northeastern China. The impact of healthcare resource allocation also exhibited marked regional variations, with the most pronounced effects observed in northeastern areas. These findings provide novel insights into understanding the spatial patterns of dysentery transmission in China and offer scientific evidence for developing targeted regional prevention and control strategies.

## Data Availability

The original contributions presented in the study are included in the article/Supplementary material, further inquiries can be directed to the corresponding author.
